# Supplementary material for: Digital technologies and performance incentives: evidence from businesses in the Swiss economy
Source: Swiss J Econ Stat. 2025 Jan 30;161(1):1. doi: 10.1186/s41937-024-00132-3 (PMC11782451; doi:10.1186/s41937-024-00132-3)
Supplement: Supplementary file 1 — Additional file 1. [file 41937_2024_132_MOESM1_ESM.pdf]

Online Appendix

A1 Theoretical framework (detailed version)

A1.1 Computer technologies and performance incentives

Starting with computer technologies (stationary and mobile ICT equipment), we can argue that these technologies are likely to help reduce the cost of organizational monitoring because they improve the measurement of employee input and output. According to Bayo-Moriones et al. (2022), computers can reduce the cost of monitoring the employees' output because electronic performance monitoring devices facilitate the measurement of employee output. However, computer technologies can also reduce the cost of monitoring the employees' input through the use of devices such as video, email monitoring, phone tapping or tracking computer content and time usage. In this way, computer technologies provide additional and more objective effort and performance data that can be analyzed. The improved measurement of employee behavior and performance should therefore encourage companies to intensify their performance measurement and thus the design of performance pay plans. Hence, we expect that computer technologies in the form of stationary and mobile ICT equipment will increase the companies' commitment in setting performance incentives.

A1.2 Business software and performance incentives

We first illustrate the theoretical connection between business software and performance incentives by the example of enterprise resource planning (ERP). ERP collects data from different areas of the company (e.g., human resources, manufacturing, finance), links this information and makes it available to executives, line managers and human resource professionals, so that these managerial employees can make data-based decisions. These decisions may relate, for example, to the detailed planning of talent management or the monitoring of worker activities. Furthermore, the ERP software analyzes the data and makes it available to managers, so that they can understand why some employees are more effective than others (Hitt et al., 2002; Aral et al., 2012). All in all, therefore, ERP systems help reduce the cost of organizational monitoring by improving the measurement of employee input and output via the provision of additional data. As a result, we expect that the use of ERP will not only encourage companies to become more involved in performance measurement, but also in performance pay.

Regarding the other types of business software, we can state that customer relationship management (CRM) software stores comprehensive data about customers and inherently

includes information about employees who interact with those customers (Aral et al., 2006). The availability of additional information about the customer-employee relationship, such as the number of interactions per period and the level of customer satisfaction, helps companies improve their performance measurement and thus reduce the cost of organizational monitoring. Furthermore, document management systems (DMS) cover aspects such as document and version control, allowing companies to track individual employees' contributions to specific projects (Alade, 2023). Finally, groupware promotes online communication and collaboration within companies as well as among organisational members and stakeholders, thereby often comprising data on employees' schedules or the number and duration of online calls (Collazos et al., 2019; Koriat and Gelbard, 2019). Overall, therefore, business software collects a number of data that make performance measurement more accurate than before. As a result, the cost of organizational monitoring can be reduced, thus intensifying both the use of performance measurement techniques and the design of pay-for-performance plans.

A1.3 Industry 4.0 key technologies and performance incentives

Finally, there are various digital technologies that can be assigned to the key technologies of Industry 4.0. The term Industry 4.0 is based on the fourth industrial revolution and reflects the digitalization, automation and networking of contemporary manufacturing. At the heart of Industry 4.0 are cyber-physical systems connecting the cyber world (information technologies) with the physical world (e.g., production technologies, products, human work) via the Internet. Instead of discussing the role of each individual Industry 4.0 technology on the prevalence of performance incentives in detail, we first restrict our theoretical analysis to three prominent examples and briefly address the other technologies present in the SES data set at the end of the section.

Starting with robotics, i.e., the use of industry, service or collaborative robots (cobots) in organizations, we can observe that robots have evolved beyond automating simple routine tasks and are capable of performing more cognitively complex work as well as tasks that require certain types of manual dexterity. They can perform many tasks such as assembly, welding, painting, packaging, picking and placing, and transportation of materials, which enables a division of labor between workers and robots, so that workers can specialize in more cognitive tasks and robots in manual tasks, thereby assisting human labor. In this way, the performance that is attributable to production workers may be easier to observe, which directly implies a reduction in the cost of organizational monitoring. In addition, robots often provide additional performance data that can be analyzed, thus also reducing the cost of organizational monitoring by helping to improve the measurement of worker

activities (Dixon et al., 2021). For these reasons, we expect that companies will increase both the level of performance measurement in the form of setting performance targets and conducting performance evaluations and the level of pay-for-performance usage.

Another key technology of Industry 4.0 to be discussed here is big data analytics combining the use of big data and artificial intelligence (AI) to analyze voluminous structured and unstructured data that stem from various sources such as company web sites, social media sites, or e-commerce platforms. In this way, the combined use of AI and big data increases the data available to monitor employees and predict their behavior (e.g., Tambe et al., 2019; Giermindl et al., 2022). A good example of big data analytics is the use of AI-based robots that support human decision-making in personnel recruitment by conducting job interviews instead of human recruiters. The idea behind the use of AI in staff recruitment is to eliminate unconscious biases human recruiters might bring to the hiring process (e.g., anchoring bias, availability bias) and to avoid discrimination in hiring with respect to personal characteristics such as gender, nationality, and age. This is more likely to succeed, the more large-scale and high-quality personnel data (e.g., texts and recordings on online job applications or skills and performance assessments) is available for the development of algorithms (Tambe et al., 2019). Hence, the use of AI and big data in personnel recruitment improves the measurement of abilities, personality traits or applicant performances and additionally increases decision quality in recruitment processes. This reduces the cost of organizational monitoring[1], which is likely to encourage companies to invest in performance measurement and pay-for-performance plans.

The next important key technology of Industry 4.0 is cloud computing and storage. Cloud computing refers to the use of IT infrastructures and services that are rented as a service and accessed via the Internet. Hence, the data is not stored on-site on local computers, but can be found at different locations (illustrated by a data cloud). In this vein, cloud storage solutions provide a cost-effective way to store, access and manage data, including information about the behavior and performance of employees at work (Brau et al., 2023; Cho et al., 2023). An important application of cloud computing is Software-as-a-Service (SaaS) (e.g., Pistorius, 2020; Frank et al., 2023). By means of this cloud service, customers receive access to certain software applications of service providers via the Internet, without installing the software on their computers and without having access to the provider's infrastructure or the operating systems. For example, the service providers Sapience Analytics and VoloMetrix offer SaaS solutions that allow companies to track the working time of their employees and see in detail how employees spend their working

[1]In the case of recruitment decisions, it would probably be more appropriate to use the term 'screening costs' rather than 'monitoring costs', as the agency problem here is pre-contractual and not post-contractual.

day (Beckmann and Gerten, 2018; SapienceAnalytics, 2021; Nyman et al., 2024). The time-tracking software provides anonymized, aggregated data on employees' time usage behavior. The software processes information extracted from emails, shared calendars, instant messenger programs and corporate social platforms, among others. The analyzed data is presented in user dashboards, enabling managers and executives to make data-driven decisions. Overall, therefore, we can conclude that time-tracking software provided as an SaaS solution generates various performance indicators that help companies to improve and simplify the measurement of employee behavior and performance, thus reducing the cost of organizational monitoring. This in turn is likely to encourage companies to increase their resources spent on performance measurement and the design of pay-for-performance plans.

The theoretical argumentation so far, neglects the empirical finding that certain digital technologies (automation technologies) lead to a substitution of labor. In particular, medium-skilled workers performing routine tasks are at risk of being automated by technologies such as robots and AI (e.g., Autor et al., 2006; Helper and Henderson, 2014; Autor, 2015; Acemoglu et al., 2016; Arntz et al., 2016; Brynjolfsson and Mitchell, 2017; Acemoglu and Restrepo, 2018; Autor and Salomons, 2018; Acemoglu and Restrepo, 2020). This literature explains the relevance of labor substitution with various comparative advantages of automation technologies over over human work (Dixon et al., 2021). First, many tasks that require physical effort can be carried out by robots with greater productivity, precision, and manual dexterity than by human workers. Second, automation technologies can carry out their tasks over long periods of time and without interruptions, breaks or rest periods (including vacations), without getting tired and without making mistakes. Automation technologies also do not go on strike, and they are not members of trade unions and therefore do not demand pay rises. Their usage is therefore often more productive and less cost-intensive than the use of human labor. Third, the use of automation technologies prevents the occurrence of agency problems resulting from information asymmetries, as automation technologies, unlike human workers, do not run the risk of acting in their own interests in the workplace. While the first argument exclusively refers to the use of robots due to their reference to the completion of physical tasks, the last two arguments do apply to both the use of robots and AI. Moreover, while robots are usually supposed to automate manual and physical demanding tasks of low- and medium-skilled blue-collar workers, AI is especially likely to replace medium-skilled white-collar workers carrying out cognitive routine tasks.

This reasoning suggests that automation technologies such as robots and AI reduce the cost of organizational monitoring not only through improving the measurement of employee behavior and performance, but also through employee substitution, which is pos-

sible because automation technologies are able to minimize human errors in a wide range of tasks, thus reducing variation in production and service processes (Dixon et al., 2021). If robots and AI take over the tasks of workers in production and administration, fewer workers will need to be monitored, which directly implies that fewer supervisors will be needed to monitor the activities of the subordinate workers. In this case, the cost of organizational monitoring can be reduced by using robots and AI to replace both the supervisors and the workers to be monitored, which also mitigates agency problems. So, if production and administration work can be replaced more and more by robots and AI, companies are likely to decrease their commitment to setting performance incentives through performance targets, performance evaluations and pay-for-performance plans.

Different arguments can also be found regarding the effects of the other key technologies of Industry 4.0 that are included in the SES data set. In some cases, the reduction in the cost of organizational monitoring seems to be achieved primarily through improved measurement of employee behavior and performance, while in other cases employee substitution seems to be the focus. For example, virtual boardrooms facilitate online meetings while collecting data about employees' work, such as time spent in meetings or the frequency of meetings including their cancellations (e.g., Gelbard et al., 2018; Giermindl et al., 2022). Even audio and video recordings are used for this (e.g., Polzer, 2022). Furthermore, the implementation of the Internet of Things (IoT) is accompanied by increased data collection through smart sensors and other IoT devices (e.g., Lee et al., 2013; Gaur et al., 2019; Katuse and Gaur, 2024). A similar argument applies to augmented reality applications, which can facilitate both the collection and the analysis of the newly gained information (e.g., Hannola et al., 2018; Ferreira et al., 2021; Arena et al., 2022). Hence, virtual boardrooms, the IoT, and augmented reality represent technologies that are likely to reduce the cost of organizational monitoring through improved measurement of employee behavior and performance, while the potential for employee substitution appears to be limited.

This could be reversed with blockchain, cyber-physical system (CPS), and additive manufacturing. Blockchain applications can, on the one hand, facilitate database management (e.g., Casino et al., 2019), but can, on the other hand, also replace employees in areas such as accounting, auditing, verification, or banking (e.g., Manski, 2017). Similarly, CPS can automate even complex information processing tasks (e.g., Windelband, 2014; Waschull et al., 2020), but they can also enable the monitoring of employees, for example through peer-to-peer comparisons (Lee et al., 2013). Finally, additive manufacturing has the potential to have a major impact on production processes, even in labor-intensive sectors such as construction, leading to a reduction in the number of workers or at least changing the structure of the workforce (e.g., Adepoju, 2022; Felice et al., 2022).

A2 Sensitivity analysis

A2.1 Alternative measures for digital technologies

Our observational SES data contain a supplementary set of digital technology variables that can be used as alternative measures to construct an analogue to the composite variable $TFint$ applied in our baseline specifications. These variables refer to the efforts and expenses that companies are incurring to meet the challenges of digital transformation. Within this context, $DTinv$ measures the investments in digital equipment as a percentage of total investments. Furthermore, $FTexp$ captures the expenses for technology-induced further training as a proportion of all training expenses made. REC is the proportion of recently recruited employees who are endowed with digital skills, such as programming, IT-development, or data analytics, measured in relation to all recently hired personnel. Finally, MOB^m and MOB^{nm} represent the proportion of managerial and non-managerial employees for whom their employer provides mobile devices capable of establishing internet connections, such as cell phones, tablet computers, or notebooks.

In analogy to the $DTint$ variable, we construct a composite measure of a company's amount of efforts and expenses to cope with the challenges of digital transformation $DTeff$, i.e.,

$$\begin{aligned} DTeff = STD\{&STD(DTinv) + STD(FTexp) + STD(REC) \\ &+ STD(MOB^m) + STD(MOB^{nm})\}\,. \end{aligned}$$

As before, $DTeff$ has zero mean and unit variance by construction.[2]

Similar to the binary treatment variable $TFint$ used in our baseline specifications to separate technology-friendly from technology-averse companies, we construct a corresponding treatment variable based on $DTeff$, thereby proceeding analogously to the variable definition described in section 3.2. After applying the respective two-step procedure and sorting the establishments into their firm size and industry cell, the treatment variable $TFeff$ is specified as

$$TFeff = \begin{cases} 1 & \text{if } DTeff > DTeff^{0.5} \\ 0 & \text{if } DTeff \leq DTeff^{0.5}\,, \end{cases}$$

where $DTeff^{0.5}$ represents the median of the $DTeff$ distribution in a specific firm size-industry cell.

[2]This measure of digital affinity is closely related to the taxonomy of digital intensive sectors as developed by Calvino et al. (2018). The authors combine indicators for ICT investment, purchases of ICT intermediates, robot use, ICT specialists, and online sales.

Table A1 presents the doubly robust ATE estimates with $TFeff$ as treatment variable. The balance statistics provided in the lower area of the table indicate a high degree of covariate balance between the treatment and control groups. The application of IPW leads to a significant reduction in the maximum ASMD, decreasing it substantially from 28.8% to 4.2%. Consequently, none of the ASMD values exceeds the threshold of 0.1 after IPW. To ensure common support, seven observations from the original sample before IPW were dropped.

Table A1: Alternative treatment variable indicating technology-friendliness

	Inc^m (1)	Inc^{nm} (2)	$Eval^m$ (3)	$Eval^{nm}$ (4)	$Target^m$ (5)	$Target^{nm}$ (6)	Pay^m (7)	Pay^{nm} (8)
$TFeff$	0.417***	0.366***	0.240**	0.166	0.320***	0.315***	0.381***	0.311**
	(0.115)	(0.118)	(0.119)	(0.104)	(0.118)	(0.121)	(0.110)	(0.121)

	Mean ASMD	Max ASMD	№ ASMD > 0.1	№ observations
Pre-weighting	0.072	0.288	5	446
Post-weighting	0.013	0.042	0	439

Source: Swiss Employer Survey (SES); own calculations.
Notes: *, **, and *** represent statistical significance at the 10%, 5%, and 1% level, respectively. Each entry in the table refers to a distinct estimation. The values in parentheses represent robust standard errors. "Mean ASMD" refers to the mean of the absolute standardized mean difference across all included covariates. "Max ASMD" shows the highest value of ASMD associated with one of the covariates. "№ ASMD > 0.1" denotes the number of covariates which have an ASMD higher than 10%. Calculations include sample weights and IPW.

The estimated ATEs using $TFeff$ as a new treatment variable turn out to be statistically significant and positive at least at the 5%-level in all specifications except for the model with $Eval^{nm}$ as outcome variable. For $Eval^{nm}$ the estimated ATE turns out to be marginally statistically insignificant ($p = 0.111$). Most importantly, however, the estimated ATEs for $TFeff$ are highly significant in the main econometric models with the composite variables Inc^m and Inc^{nm} as performance incentives outcome variables. The effect sizes corresponding to the different hierarchical levels are also again relatively similar. Overall, the empirical findings in this section confirm the notion that the improved measurement effect dominates the employee substitution effect and provides additional evidence for the validity of *Hypothesis* 2 a).

A2.2 ATE estimates for technological leaders and laggards

The construction of the composite measure for technological affinity, denoted as $TFint$, relies on dichotomizing a continuous variable that contains information about the technological status of companies. In our baseline specifications, we opt for the median of the continuous technology variable as a natural threshold for separating technology-friendly

from technology-averse businesses. Figure 4 in section 3.5.2 indicates that this choice is definitely appropriate given the data available. However, to further assess whether or not the binarization of a continuous variable is appropriate, we need to consider alternative thresholds to separate the treatment from the control group. The binarization of a continuous technology variable appears to be adequate if the estimated ATEs resulting from the use of the adjusted treatment variables turn out to be reasonable and economically plausible with regard to magnitude and statistically significance. Hence, with this robustness check, we also aim at ensuring that our ATE estimates for technological affinity are reliable and not driven by the choice of a specific threshold.

Our approach of creating alternative measures indicating different degrees of technological affinity is inspired by Acemoglu et al. (2007, pp. 1759, 1760) who introduce a concept of technological leadership by separating firms that are positioned "*closer to the technological frontier*" from firms that are located further away from the technological frontier. Following the concept of technological leadership, we define firms as technological leaders if they surpass the 75th percentile in the distribution of $DFint$. To operationalize this concept, we again divide our sample into six different cells, taking into account both company size and industry type. Within each of these cells, we identify technological leaders ($TLeader$) as firms belonging to the upper quartile (top 25%) of the $DTint$ distribution, i.e.,

$$TLeader = \begin{cases} 1 & \text{if } DTint > DTint^{0.75} \\ 0 & \text{if } DTint \leq DTint^{0.75} \end{cases},$$

where $DTint^{0.75}$ represents the 75th percentile of the $DTint$ distribution in a respective cell. We apply an analogous procedure to identify technological laggards ($TLaggard$), which we define as firms belonging to the lower quartile (bottom 25%) of the $DTint$ distribution within a specific cell. Hence, $TLaggard$ is generated as

$$TLaggard = \begin{cases} 1 & \text{if } DTint \leq DTint^{0.25} \\ 0 & \text{if } DTint > DTint^{0.25} \end{cases},$$

where $DTint^{0.25}$ represents the 25th percentile of the $DTint$ distribution in a respective cell. We expect negative ATEs for $TLaggard$ and positive ATEs for $TLeader$. This would be consistent with figure 4 and our baseline ATE estimates.

Table A2 presents the doubly robust ATE estimates and the balance statistics for the specifications with $TLeader$ and $TLaggard$ as treatment variables. In terms of the balance diagnostics, IPW appears to be effective in both treatment model specifications. For $TLeader$, none of the ASMDs exceeds the threshold of 0.1 after IPW, while prior to IPW

seven covariates exceeded the 0.1 threshold. In the case of $TLaggards$, after IPW, three covariates still have ASMD values slightly beyond the 10% mark. However, the exceedings are relatively modest, with the highest ASMD reaching 12.9%. Therefore, we assess the overall achieved covariate balance for both treatment variables $TLeader$ and $TLaggard$ as satisfactory.

Table A2: Technological leaders and technological laggards

	Inc^m (1)	Inc^{nm} (2)	$Eval^m$ (3)	$Eval^{nm}$ (4)	$Target^m$ (5)	$Target^{nm}$ (6)	Pay^m (7)	Pay^{nm} (8)
$TLeader$	0.461*** (0.122)	0.463*** (0.127)	0.379*** (0.121)	0.369*** (0.103)	0.337*** (0.125)	0.315** (0.130)	0.325** (0.132)	0.319** (0.147)

	Mean ASMD	Max ASMD	№ ASMD > 0.1	№ observations
Pre-weighting	0.088	0.308	7	446
Post-weighting	0.027	0.087	0	438

	Inc^m (1)	Inc^{nm} (2)	$Eval^m$ (3)	$Eval^{nm}$ (4)	$Target^m$ (5)	$Target^{nm}$ (6)	Pay^m (7)	Pay^{nm} (8)
$TLaggard$	-0.660*** (0.125)	-0.684*** (0.118)	-0.625*** (0.135)	-0.612*** (0.119)	-0.433*** (0.122)	-0.478*** (0.123)	-0.433*** (0.103)	-0.391*** (0.109)

	Mean ASMD	Max ASMD	№ ASMD > 0.1	№ observations
Pre-weighting	0.099	0.343	7	446
Post-weighting	0.032	0.129	3	440

Source: Swiss Employer Survey (SES); own calculations.
Notes: *, **, and *** represent statistical significance at the 10%, 5%, and 1% level, respectively. Each entry in the table refers to a distinct estimation. The values in parentheses represent robust standard errors. "Mean ASMD" refers to the mean of the absolute standardized mean difference across all included covariates. "Max ASMD" shows the highest value of ASMD associated with one of the covariates. "№ ASMD > 0.1" denotes the number of covariates which have an ASMD higher than 10%. Calculations include sample weights and IPW.

Looking at the estimated ATEs, it can be noted that the effects for $TLeader$ are positive and statistically significant at least at the 5% level, in the main specifications with Inc^m and Inc^{nm} as outcome variables even at the 1% level. However, it is noticeable that the estimated ATEs in all specifications are slightly smaller than the ATEs in the corresponding baseline specifications with $TFint$ as treatment variable. This finding is consistent with the relatively flat performance incentives curve to the right of the median, as displayed in figure 4. As anticipated, the doubly robust ATEs for $TLaggard$ are highly significant with a negative sign in all performance incentives specifications. Again, for both treatment variables $TLeader$ and $TLaggard$, no clear divergence in the effects between hierarchical levels are visible. In summary, therefore, we can conclude that the binarization of our continuous variable $DTint$, which measures the intensity of a company's use of various digital technologies, does not pose any methodological problems.

A2.3 Inverse probability weighting

Applying conventional IPW requires a correctly specified treatment model (3) and no unobserved counfounders (assumption A2). Given that the weighting of observables via the inverse of the propensity score sufficiently controls for confounding and that the treatment model (3) for estimating the propensity score and weights is correctly specified, the IPW estimator provides consistent estimates of the ATE (Funk et al., 2011). The estimation results based on IPW are displayed in table A3.

Table A3: ATE estimation using inverse probability weighting (IPW)

	Inc^m (1)	Inc^{nm} (2)	$Eval^m$ (3)	$Eval^{nm}$ (4)	$Target^m$ (5)	$Target^{nm}$ (6)	Pay^m (7)	Pay^{nm} (8)
$TFint$	0.646***	0.726***	0.468***	0.538***	0.495***	0.584***	0.497***	0.448***
	(0.124)	(0.125)	(0.130)	(0.124)	(0.123)	(0.120)	(0.120)	(0.128)

Source: Swiss Employer Survey (SES); own calculations.
Notes: *, **, and *** represent statistical significance at the 10%, 5%, and 1% level, respectively. Each entry in the table refers to a distinct estimation. The values in parentheses represent robust standard errors. Since there are no changes in the weighting procedure compared to the doubly robust estimator, the balance statistics reported in table A7 continue to apply. Calculations include sample weights and IPW.

The ATEs estimated by IPW turn out to be very similar to our baseline ATE estimates obtained by applying the doubly robust estimator. This is true for both the magnitude and significance of the estimated ATEs. Similar IPW and doubly robust ATE estimates suggest that at least the treatment model (3) is correctly specified in the doubly robust ATE estimation approach. According to the doubly robust property, therefore, our ATE estimates obtained from the doubly robust estimator turn out to be consistent, unless there are unobserved confounders that are correlated with the treatment and outcome variables.

A2.4 Adjustments to the trimming and weighting procedures

The baseline specification of the present study adopts a relatively aggressive trimming approach, utilizing cut-off values at the 5th and 95th percentiles. However, as discussed in section 4.2, there is significant flexibility in determining the extent of trimming. Therefore, it is crucial to provide evidence that the obtained estimation results are not predominantly affected by a specific use of the computed weights. In other words, as suggested by Solon et al. (2015), researchers employing weighting schemes should report and compare both weighted and unweighted estimation results and engage in discussion of the disparities between them.

Table A4: Doubly robust ATE estimates: adjusted sample weights and trimming

No sample weights	Inc^{m} (1)	Inc^{nm} (2)	$Eval^{m}$ (3)	$Eval^{nm}$ (4)	$Target^{m}$ (5)	$Target^{nm}$ (6)	Pay^{m} (7)	Pay^{nm} (8)
$TFint$	0.493***	0.545***	0.391***	0.412***	0.383***	0.469***	0.341***	0.299***
	(0.087)	(0.087)	(0.091)	(0.088)	(0.092)	(0.091)	(0.090)	(0.095)
	Mean ASMD		Max ASMD		№ ASMD > 0.1		№ observations	
Post-weighting	0.007		0.023		0		440	
**No trimming**	Inc^{m} (1)	Inc^{nm} (2)	$Eval^{m}$ (3)	$Eval^{nm}$ (4)	$Target^{m}$ (5)	$Target^{nm}$ (6)	Pay^{m} (7)	Pay^{nm} (8)
$TFint$	0.621***	0.743***	0.410***	0.506***	0.453***	0.608***	0.538***	0.493***
	(0.115)	(0.114)	(0.130)	(0.106)	(0.118)	(0.115)	(0.112)	(0.128)
	Mean ASMD		Max ASMD		№ ASMD > 0.1		№ observations	
Post-weighting	0.013		0.064		0		440	
**1st & 99th Perc.**	Inc^{m} (1)	Inc^{nm} (2)	$Eval^{m}$ (3)	$Eval^{nm}$ (4)	$Target^{m}$ (5)	$Target^{nm}$ (6)	Pay^{m} (7)	Pay^{nm} (8)
$TFint$	0.634***	0.749***	0.434***	0.519***	0.477***	0.625***	0.521***	0.478***
	(0.116)	(0.113)	(0.129)	(0.106)	(0.117)	(0.114)	(0.111)	(0.126)
	Mean ASMD		Max ASMD		№ ASMD > 0.1		№ observations	
Post-weighting	0.017		0.095		0		441	
**2.5th & 97.5th Perc.**	Inc^{m} (1)	Inc^{nm} (2)	$Eval^{m}$ (3)	$Eval^{nm}$ (4)	$Target^{m}$ (5)	$Target^{nm}$ (6)	Pay^{m} (7)	Pay^{nm} (8)
$TFint$	0.644***	0.741***	0.450***	0.526***	0.500***	0.621***	0.505***	0.456***
	(0.115)	(0.113)	(0.125)	(0.106)	(0.116)	(0.114)	(0.110)	(0.123)
	Mean ASMD		Max ASMD		№ ASMD > 0.1		№ observations	
Post-weighting	0.017		0.075		0		441	

Source: Swiss Employer Survey (SES); own calculations.
Notes: *, **, and *** represent statistical significance at the 10%, 5%, and 1% level, respectively. Each entry in the table refers to a distinct estimation. The values in parentheses represent robust standard errors. "Mean ASMD" refers to the mean of the absolute standardized mean difference across all included covariates. "Max ASMD" shows the highest value of ASMD associated with one of the covariates. "№ ASMD > 0.1" denotes the number of covariates which have an ASMD higher than 10%. Calculations include sample weights and IPW.

In this context, table A4 provides the doubly robust ATE estimates and the ASMD balance diagnostics resulting from regressions with the composite dummy variable $TFint$ as treatment variable. The upper panel in table A4 reports a specification without sample weights but with trimming at the 5th and 95th percentiles. The second panel displays a specification without trimming but with the application of sample weights. Finally, the third and lower panels show specifications with both sample weights and trimming at cut-off values at the 1st and 99th percentiles (panel 3) and at the 2.5th and 97.5th percentiles (lower panel). In each of the four panels, we observe convincing balance statistics. After IPW, neither the mean ASMD nor the maximum ASMD have values greater than 0.1. Therefore, we do not find a single covariate imbalance in any of the specifications. This finding is consistent with the corresponding balance statistics obtained in our baseline specification (see table A7) and indicates a high quality of the respective model specification, which is independent of the applied sample weights-trimming combination.

Moreover, we can see that each of the estimated doubly robust ATEs for $TFint$ depicted in table A4 turns out to be (highly) statistically significant with a positive sign, thus confirming the significant ATE estimates resulting from our baseline specifications using sample weights and trimming at cut-off values at the 5th and 95th percentiles. This maintained statistical significance suggests that our decisions regarding weighting characteristics are not the driving force behind the obtained statistically significant ATE estimates. With respect to magnitude, the ATEs are slightly smaller than the corresponding baseline ATE estimates when the sample weights are omitted and tend to be slightly higher in the other three trimming approaches. Looking simultaneously at Inc^m and Inc^{nm}, the most pronounced effect size differences in ATEs can be observed when comparing the baseline specifications with the specifications without sample weights reported in the upper panel of table A4.

A2.5 Data-driven selection of covariates

In the selection of control variables to be used in our baseline regression models, we were guided by economic theory. In this sensitivity check, we rely on a pure data-driven approach to select the control variables for our treatment model (3) and the regression models for the potential outcomes (1) and (2), thereby focusing on the statistical requirements for specifying a set of covariates in regression models based on the estimation of ATEs. Here, we apply the double-selection lasso[3] linear regression approach in which lassos are used to select the appropriate covariates. Specifically, we let lasso select a set of covariates that is (a) correlated with either the treatment variable (instruments) or the

[3]Lasso is the abbreviation for least absolute shrinkage and selection operator.

outcome variable (potential confounders, prognostically important covariates), (b) correlated with both the treatment and outcome variables (true confounders). Lasso selects the variables described under (a) and (b) from the full set of variables included in our firm-level data set.

Table A5: Doubly robust ATE estimates with lasso selected potential confounders and instruments

	Inc^m (1)	Inc^{nm} (2)	$Eval^m$ (3)	$Eval^{nm}$ (4)	$Target^m$ (5)	$Target^{nm}$ (6)	Pay^m (7)	Pay^{nm} (8)
$TFint$	0.535***	0.595***	0.421***	0.458***	0.476***	0.503***	0.322***	0.309***
	(0.104)	(0.105)	(0.116)	(0.102)	(0.107)	(0.110)	(0.108)	(0.113)
№ control variables	45	45	42	43	40	43	43	41
№ observations	433	429	436	435	436	435	434	434
Pre-weighting								
Mean ASMD	0.078	0.077	0.081	0.079	0.081	0.078	0.079	0.080
Highest ASMD	0.339	0.339	0.339	0.339	0.339	0.339	0.339	0.339
№ ASMD > 0.1	13	13	13	13	13	13	13	13
Post-weighting								
Mean ASMD	0.024	0.022	0.025	0.023	0.023	0.023	0.024	0.024
Highest ASMD	0.123	0.118	0.113	0.116	0.123	0.116	0.115	0.119
№ ASMD > 0.1	2	1	2	1	1	2	1	1

Source: Swiss Employer Survey (SES); own calculations.
Notes: *, **, and *** represent statistical significance at the 10%, 5%, and 1% level, respectively. Each entry in the table refers to a distinct estimation. The values in parentheses represent robust standard errors. "Mean ASMD" refers to the mean of the absolute standardized mean difference across all included covariates. "Max ASMD" shows the highest value of ASMD associated with one of the covariates. "№ ASMD > 0.1" denotes the number of covariates which have an ASMD higher than 10%. "№ control variables" denotes the number of control variables the double-selection lasso procedure chooses for the respective ATE estimation. Calculations include sample weights and IPW. Table A8 depicts the full regression results for the dependent variables Inc^m and Inc^{nm}.

The double-selection algorithm proceeds in three steps. The first step is a lasso linear regression of the treatment variable DT on all variables X^{all} that are available in our data set.[4] The second step involves lasso linear regressions of the potential outcome variables PI_1^j and PI_0^j on X^{all}. The algorithm selects the statistically significant variables in X^{all} from both steps to be used as the data-driven set of control variables X^{lasso}. The final step is the re-estimation of the baseline model, where X is replaced by X^{lasso}. The doubly robust ATE estimates resulting from data-driven covariates selection are displayed in tables A5 and A6. As before, we show the doubly robust ATE estimates for our main treatment variable $TFint$.

First of all, we can note that lasso determines between 40 and 45 variables as control

[4] A list and descriptive statistics for the additionally included control variables are available in table A10.

Table A6: Doubly robust ATE estimates with lasso selected true confounders

	Inc^m (1)	Inc^{nm} (2)	$Eval^m$ (3)	$Eval^{nm}$ (4)	$Target^m$ (5)	$Target^{nm}$ (6)	Pay^m (7)	Pay^{nm} (8)
$TFint$	0.450***	0.539***	0.346***	0.399***	0.396***	0.458***	0.331***	0.260**
	(0.115)	(0.118)	(0.127)	(0.111)	(0.115)	(0.114)	(0.111)	(0.110)
№ control variables	20	16	13	14	13	14	16	9
№ observations	429	434	431	436	437	442	417	440
Pre-weighting								
Mean ASMD	0.095	0.101	0.095	0.107	0.110	0.120	0.091	0.093
Highest ASMD	0.339	0.339	0.339	0.339	0.339	0.339	0.339	0.314
№ ASMD > 0.1	8	7	6	6	6	6	6	3
Post-weighting								
Mean ASMD	0.016	0.027	0.010	0.016	0.020	0.022	0.011	0.030
Highest ASMD	0.069	0.090	0.054	0.066	0.085	0.075	0.039	0.087
№ ASMD > 0.1	0	0	0	0	0	0	0	0

Source: Swiss Employer Survey (SES); own calculations.
Notes: *, **, and *** represent statistical significance at the 10%, 5%, and 1% level, respectively. Each entry in the table refers to a distinct estimation. The values in parentheses represent robust standard errors. "Mean ASMD" refers to the mean of the absolute standardized mean difference across all included covariates. "Max ASMD" shows the highest value of ASMD associated with one of the covariates. "№ ASMD > 0.1" denotes the number of covariates which have an ASMD higher than 10%. "№ control variables" denotes the number of control variables the double-selection lasso procedure chooses for the respective ATE estimation. Calculations include sample weights and IPW. Table A8 depicts the full regression results for the dependent variables Inc^m and Inc^{nm}.

variables for the 'generous' model specification, in which the potential confounders as well as the instruments should be selected, while between 9 and 20 true confounders are selected for the 'more restrictive' model specification. The number of variables selected is thus consistent in that we use a number of theory-driven control variables in our baseline model that is pretty much in the middle of the 'restrictive' and the 'generous' versions of variable selection, namely 22. Furthermore, the balance statistics reported in both tables document that IPW significantly improves the mean and highest ASMDs. While in table A5 (table A6) 13 (3-8) covariates prior to IPW have an $ASMD > 0.1$, this number reduces to one to two variables after IPW in table A5, with the highest ASMD not exceeding a value of 0.123 across all specifications, and to zero covariates for the true confounders specification shown in table A6. Finally, across all model specifications, the estimated ATEs from tables A5 and A6 turn out to be somewhat smaller than the corresponding ATE estimates resulting from our baseline models. However, they do not lose any significance but remain statistically highly significant in most cases. Overall, the results of this robustness check with a data-driven selection of control variables strongly confirm our baseline ATE estimates presented in section 5.2.

A3 Supplementary tables

Table A7: Covariate balance diagnostics (ASMD) per treatment variable

	TFint	
	Pre	Post
Mean ASMD	0.081	0.019
Highest ASMD	0.314	0.079
№ ASMD > 0.1	7	0
№ observations	446	439

	NonStat		Stat		Groupware		ERP	
	Pre	Post	Pre	Post	Pre	Post	Pre	Post
Mean ASMD	0.122	0.101	0.097	0.082	0.145	0.054	0.107	0.028
Highest ASMD	0.462	0.466	0.354	0.231	0.430	0.200	0.320	0.118
№ ASMD > 0.1	12	7	10	8	12	3	9	2
№ observations	446	377	446	361	446	408	446	424

	DMS		CRM		MIS		AI/BigData	
	Pre	Post	Pre	Post	Pre	Post	Pre	Post
Mean ASMD	0.097	0.019	0.085	0.017	0.064	0.036	0.082	0.019
Highest ASMD	0.488	0.065	0.342	0.043	0.133	0.112	0.273	0.091
№ ASMD >0.1	6	0	8	0	5	2	7	0
№ observations	446	430	446	441	446	442	446	445

	CPS		IoT		Cloud		VirtBoard	
	Pre	Post	Pre	Post	Pre	Post	Pre	Post
Mean ASMD	0.071	0.029	0.138	0.062	0.028	0.013	0.082	0.061
Highest ASMD	0.306	0.100	0.632	0.252	0.194	0.050	0.274	0.218
№ ASMD > 0.1	4	0	11	4	1	0	6	3
№ observations	446	444	446	404	446	444	446	399

	Robotics		AddMan		AugReality		Block	
	Pre	Post	Pre	Post	Pre	Post	Pre	Post
Mean ASMD	0.134	0.119	0.108	0.117	0.190	0.159	0.274	0.151
Highest ASMD	0.393	0.453	0.420	0.456	0.674	0.484	1.085	0.529
№ ASMD > 0.1	10	9	9	9	12	12	16	11
№ observations	446	429	446	412	446	356	446	140

Source: Swiss Employer Survey (SES); own calculations.
Notes: "Mean ASMD" refers to the mean of the absolute standardized mean differences across all included covariates. "Max ASMD" shows the highest value of ASMD associated with one of the covariates. "№ ASMD > 0.1" denotes the number of covariates which have an ASMD higher than 10%. "Pre" refers to the statistics evaluated prior to IPW, while "Post" refers to the same statistics after IPW and enforcing common support. Table A11 introduces the abbreviations. Calculations include sample weights. All weights are trimmed at the 5th and 95th percentiles.

Table A8: Overview: Full regression results

	Baseline		Lasso		Lasso: true confounders	
	Inc^{m}	Inc^{nm}	Inc^{m}	Inc^{nm}	Inc^{m}	Inc^{nm}
	(1)	(2)	(3)	(4)	(5)	(6)
$TFint$	0.625***	0.707***	0.535***	0.595***	0.450***	0.539***
	(0.112)	(0.111)	(0.104)	(0.105)	(0.115)	(0.118)
Competitive pressure	-0.010	0.026	-0.011	0.014	0.003	
	(0.040)	(0.036)	(0.036)	(0.033)	(0.039)	
Location: Espace Mittelland	-0.306*	0.162	0.117	0.220		
	(0.179)	(0.215)	(0.156)	(0.148)		
Location: Région lemanique	-0.325*	-0.070	0.945***	0.921***		
	(0.178)	(0.235)	(0.274)	(0.274)		
Location: Nothwestern Sw.	-0.038	0.307				
	(0.195)	(0.233)				
Location: Eastern Sw.	-0.236	0.282				
	(0.204)	(0.261)				
Location: Ticino	-0.787***	-0.916***	0.574**	0.191		-0.804***
	(0.173)	(0.246)	(0.260)	(0.264)		(0.184)
Location: Central Sw.	-0.283	0.237				
	(0.237)	(0.218)				
Location: Zurich			0.029	-0.153		
			(0.161)	(0.162)		
Capital company	0.064	-0.067				
	(0.143)	(0.139)				
Works council	-0.185	-0.086	-0.186	-0.021		
	(0.117)	(0.114)	(0.119)	(0.111)		
Legally independent	-0.193	-0.043				
	(0.124)	(0.126)				
Strategy: Expansion	0.365***	0.201*				
	(0.115)	(0.117)				
Strategy: Reduction	0.175	0.113				
	(0.164)	(0.161)				
Decision-rights assignment	-0.069	-0.044	-0.022	0.049	-0.065	
	(0.058)	(0.064)	(0.057)	(0.054)	(0.062)	
Small establishment	-0.614***	-0.329*	-0.386***	-0.302**	-0.412***	
	(0.192)	(0.182)	(0.122)	(0.119)	(0.102)	
Medium establishment	-0.256	-0.047				
	(0.190)	(0.182)				
Large establishment			0.041	-0.415*		
			(0.254)	(0.250)		
% High skilled employees	0.325	0.930*	0.121	0.347		
	(0.456)	(0.480)	(0.363)	(0.359)		
% Medium skilled employees	0.050	0.589	-0.116	0.277		
	(0.450)	(0.468)	(0.345)	(0.311)		
% Low skilled employees	-0.087	0.075	0.009	-0.059		-0.359*
	(0.474)	(0.485)	(0.363)	(0.343)		(0.215)
Service sector	0.183	0.275**				
	(0.129)	(0.132)				
Wave	-0.143	-0.129	-0.237**	-0.183*		
	(0.112)	(0.112)	(0.112)	(0.103)		
Constant	0.125	-0.993*	1.763***	-1.719***	-0.809**	-0.633**
	(0.503)	(0.515)	(0.640)	(0.596)	(0.376)	(0.287)

Continued on the next page...

...Table A8 continued

	Baseline		Lasso		Lasso: true confounders	
	Inc^m	Inc^{nm}	Inc^m	Inc^{nm}	Inc^m	Inc^{nm}
	(1)	(2)	(3)	(4)	(5)	(6)
Culture: Performance			0.086	0.074	0.123*	0.115*
			(0.067)	(0.063)	(0.065)	(0.063)
Culture: Focus on employees			0.008	-0.015		
			(0.078)	(0.081)		
Culture: Competition			-0.030	-0.095*	-0.099*	-0.147***
			(0.054)	(0.052)	(0.058)	(0.053)
Culture: Innovation			0.083	0.086	0.064	0.050
			(0.055)	(0.056)	(0.056)	(0.059)
Culture: Precision			-0.006	-0.007		
			(0.077)	(0.078)		
Strategy: Internal expansion			0.166	0.058	0.199	0.169
			(0.114)	(0.119)	(0.126)	(0.120)
Strategy: External expansion			0.200	0.029	0.172	
			(0.130)	(0.107)	(0.137)	
Strategy: Sale			0.210	0.133		
			(0.284)	(0.264)		
Strategy: Outsourcing			0.022	0.079	0.160	
			(0.173)	(0.163)	(0.184)	
Vacant position: Total			-0.001	0.004		
			(0.007)	(0.009)		
Vacant position: Low-skilled jobs			-0.003	-0.012		
			(0.013)	(0.014)		
NOGA class: D			0.762***	0.770*	0.368**	1.098***
			(0.278)	(0.446)	(0.181)	(0.381)
NOGA class: E			0.330	0.219	-1.244***	0.050
			(0.897)	(1.031)	(0.363)	(0.944)
NOGA class: F			-0.031	-0.282	-0.059	-0.391*
			(0.173)	(0.202)	(0.184)	(0.207)
NOGA class: G			0.439**	0.414**	0.455	0.316
			(0.216)	(0.210)	(0.300)	(0.254)
NOGA class: H			0.152	0.280	0.205	0.273
			(0.427)	(0.363)	(0.353)	(0.319)
NOGA class: I			0.107	-0.197		-0.328*
			(0.193)	(0.217)		(0.185)
NOGA class: J			0.532**	0.590*	0.477*	0.550*
			(0.235)	(0.319)	(0.264)	(0.332)
NOGA class: K			0.637**	0.663**		
			(0.315)	(0.256)		
NOGA class: M			0.500**	0.597***	0.437**	0.398**
			(0.215)	(0.226)	(0.189)	(0.199)
NOGA class: N			0.139	0.160		
			(0.242)	(0.284)		
NOGA class: P			-0.298	-0.049		
			(0.306)	(0.246)		
NOGA class: Q			-0.080	-0.013	-0.169	
			(0.223)	(0.187)	(0.168)	
NOGA class: R			-0.322	-0.133	-0.586*	
			(0.317)	(0.300)	(0.347)	
NOGA class: S			0.027	0.176		
			(0.271)	(0.221)		

Continued on the next page...

...Table A8 continued

	Baseline		Lasso		Lasso: True confounders	
	Inc^m	Inc^{nm}	Inc^m	Inc^{nm}	Inc^m	Inc^{nm}
	(1)	(2)	(3)	(4)	(5)	(6)
Legal form: Private company			-0.378	-0.392		
			(0.294)	(0.356)		
Legal form: Stock corperation			-0.033			
			(0.175)			
Legal form: Limited liability company			-0.203	-0.182		
			(0.243)	(0.221)		
Legal form: Other				0.251		
				(0.217)		
Legal Type: Headquarter			-0.057	-0.200		
			(0.142)	(0.126)		
Legal type: Dependant organization			0.154	0.117	0.284*	0.265*
			(0.171)	(0.155)	(0.168)	(0.156)
Legal type: None of the above			0.511**	0.281	0.642***	0.267
			(0.216)	(0.219)	(0.225)	(0.241)
German speaking			1.112***	1.152***		
			(0.228)	(0.202)		

Source: Swiss Employer Survey (SES); own calculations.
Notes: *, **, and *** represent statistical significance at the 10%, 5%, and 1% level, respectively. The values in parentheses represent robust standard errors. The number of observations and balance statistics are denoted in table A7, A5, and A6, respectively. Calculations include sample weights and IPW.

Table A9: Descriptive statistics of the included variables

Technology Variables	Mean			SD	Minimum	Maximum
	Total	Survey	Web	Total	Total	Total
$TFint$	0.500	0.522	0.456	0.501	0	1
$TLeff$	0.500	0.522	0.456	0.501	0	1
$TLeader$	0.253	0.239	0.282	0.435	0	1
$TLaggard$	0.271	0.256	0.302	0.445	0	1
Stat	0.933	0.933	0.933	0.251	0	1
NonStat	0.960	0.970	0.940	0.197	0	1
AI/BigData	0.312	0.303	0.329	0.464	0	1
ERP	0.623	0.670	0.530	0.485	0	1
DMS	0.547	0.562	0.517	0.498	0	1
MIS	0.240	0.229	0.262	0.428	0	1
CRM	0.498	0.468	0.557	0.501	0	1
Groupware	0.803	0.835	0.738	0.398	0	1
VirtBoard	0.161	0.168	0.148	0.368	0	1
Cloud	0.650	0.646	0.658	0.477	0	1
CPS	0.267	0.273	0.255	0.443	0	1
IoT	0.166	0.175	0.148	0.372	0	1
Robotics	0.123	0.114	0.141	0.329	0	1
AddMan	0.078	0.077	0.081	0.269	0	1
AugReality	0.040	0.034	0.054	0.197	0	1
Block	0.013	0.010	0.020	0.115	0	1

Continued on the next page...

...Table A9 continued

Dependent variables	Mean Total	Mean Survey	Mean Web	SD Total	Minimum Total	Maximum Total
Inc^m	0.000	0.047	-0.094	1.000	-1.529	1.656
Inc^{nm}	0.000	0.047	-0.093	1.000	-1.676	1.985
$Eval^m$ (in %)	71%	74%	66%	43.199	0	100
$Eval^{nm}$ (in %)	78%	80%	73%	37.858	0	100
$Target^m$ (in %)	57%	57%	56%	46.895	0	100
$Target^{nm}$ (in %)	52%	51%	53%	45.688	0	100
Pay^m (in %)	21%	23%	19%	36.409	0	100
Pay^{nm} (in %)	14%	16%	11%	32.340	0	100
Control variables						
Competitive pressure	3.027	2.983	3.114	1.559	1	5
Location: Espace Mittelland	0.238	0.236	0.242	0.426	0	1
Location: Région lemanique	0.128	0.128	0.128	0.334	0	1
Location: Nothwestern Sw.	0.159	0.148	0.181	0.366	0	1
Location: Eastern Sw.	0.135	0.128	0.148	0.342	0	1
Location: Ticino	0.083	0.094	0.060	0.276	0	1
Location: Central Sw.	0.099	0.091	0.114	0.299	0	1
Location: Zurich	0.159	0.175	0.128	0.366	0	1
Capital company	0.709	0.684	0.758	0.455	0	1
Works council	0.309	0.327	0.275	0.463	0	1
Legally independent	0.791	0.771	0.832	0.407	0	1
Strategy: Expansion	0.556	0.569	0.530	0.497	0	1
Strategy: Reduction	0.155	0.138	0.188	0.362	0	1
Decision-rights assignment	0.000	-0.028	0.055	1.000	-4.779	2.163
Small establishment	0.462	0.424	0.537	0.499	0	1
Medium establishment	0.426	0.465	0.349	0.495	0	1
Large establishment	0.112	0.111	0.114	0.316	0	1
% High skilled employees	0.237	0.247	0.216	0.233	0	1
% Medium skilled employees	0.488	0.498	0.468	0.249	0	1
% Low skilled employees	0.187	0.175	0.209	0.225	0	0.981
Service sector	0.646	0.636	0.664	0.479	0	1
Wave	0.334	0.000	1.000	0.472	0	1

Source: Swiss Employer Survey (SES); own calculations.
Notes: The table depicts the mean of the overall sample (*Total*), as well as of the two subsamples of establishments whose contact information we received from the Swiss Federal Statistical Office (*Survey*) and via web scraping (*Web*). SD denotes the standard deviation, while Minimum and Maximum denote the respective lowest and highest values present in the data set.

Table A10: Additional control variables included in the lasso estimations

	Mean	Standard deviation	Minimum	Maximum
Culture: Performance	3.908	0.891	1	5
Culture: Focus on employees	4.348	0.727	2	5
Culture: Competition	2.489	1.155	1	5
Culture: Innovation	3.390	1.010	1	5
Culture: Precision	4.404	0.724	1	5
Strategy: Internal expansion	0.464	0.499	0	1
Strategy: External expansion	0.262	0.440	0	1
Strategy: Sale	0.056	0.230	0	1
Strategy: Outsourcing	0.123	0.329	0	1
Vacant positions: Total	4.909	14.266	0	200
Vacant positions: Low-skilled jobs	1.149	6.319	0	120
German-speaking	0.724	0.447	0	1
French-speaking	0.193	0.395	0	1
NOGA class: C	0.200	0.400	0	1
NOGA class: D	0.011	0.105	0	1
NOGA class: E	0.007	0.082	0	1
NOGA class: F	0.137	0.344	0	1
NOGA class: G	0.065	0.247	0	1
NOGA class: H	0.020	0.141	0	1
NOGA class: I	0.087	0.283	0	1
NOGA class: J	0.034	0.180	0	1
NOGA class: K	0.054	0.226	0	1
NOGA class: L	0.027	0.162	0	1
NOGA class: M	0.072	0.258	0	1
NOGA class: N	0.063	0.243	0	1
NOGA class: P	0.025	0.155	0	1
NOGA class: Q	0.141	0.349	0	1
NOGA class: R	0.025	0.155	0	1
NOGA class: S	0.034	0.180	0	1
Legal form: Private company	0.025	0.155	0	1
Legal form: Stock corperation	0.639	0.481	0	1
Legal form: Limited liability company	0.070	0.255	0	1
Legal form: Association/foundation	0.157	0.364	0	1
Legal form: Other	0.081	0.273	0	1
Legal type: Head quarter	0.242	0.429	0	1
Legal type: Dependant organization	0.139	0.346	0	1
Legal type: Other	0.070	0.255	0	1

Source: Swiss Employer Survey (SES); own calculations.
Notes: The lasso estimation excludes the following control variables from the baseline estimation, as it includes finer grained information on these topics: Capital company, legally independent, expansion, reduction, sector.

Table A11: Overview of the surveyed digital technologies

Technology	Abbreviation	Examples
**Computer technologies**		
Non-stationary IT-equipment	NonStat	e.g., smartphones, tablets, notebooks
Stationary IT-equipment	Stat	e.g., PCs, electronic cash registers, CAD systems
**Business software**		
Groupware/Collaborative applications	Groupware	e.g., Slack, MS Teams, Zoom, Webex
Enterprise resource planning	ERP	e.g., SAP, Net Suite, MS Dynamics
Document management systems	DMS	e.g., MS Sharepoint, Rubex
Customer relationship management	CRM	e.g., Salesforce, Oracle
Management information system	MIS	e.g., Clarity Professional MIS
**Key technologies of Industry 4.0**		
Software or algorithms for IT-based process optimization	AI/BigData	e.g., artificial intelligence, big data analytics
Networking and control of machines and plants via the internet (cyber-physical systems)	CPS	e.g. smart grid, autonomous automobile systems, medical monitoring
Internet of Things	IoT	e.g., IoT-sensors, RFID chips, e-grains, NFC
Cloud storage/computing	Cloud	e.g., AWS, MS Azure, IBM Cloud, Google Cloud
Virtual boardrooms	VirtBoard	e.g., Sherpany, Dilligent Boards, iDeals, BoardEffect
Robotics, automated transport or production systems	Robotics	e.g., industrial, service or mobile robots, drones
Additive manufacturing processes	AddMan	e.g., 3D printing
Virtual/augmented reality	AugReality	e.g., MS HoloLens
Blockchain (distributed ledger technology)	Block	e.g., cryptocurrencies, non-fungible tokens, smart contracts

Source: Swiss Employer Survey (SES).

Table A12: Survey questions for the main variables

Survey question	Response options
In the following, you find a list of various technologies. Please specify, whether the respective technology is used in your company.	
	□ Stationary IT-equipment (e.g., PCs, electronic cash registers, CAD systems)
	□ Non-stationary IT equipment (e.g., smartphones, tablets, notebooks)
	□ Software or algorithms for IT-based business process optimization (e.g., artificial intelligence, big data analytics)
	□ Enterprise Resource Planning (ERP; e.g., SAP, Net Suite, MS Dynamics)
	□ Document Management System (DMS; e.g., MS Sharepoint, Rubex)
	□ Management Information System (e.g., Clarity Professional MIS)
	□ Customer Relationship Management (e.g., Salesforce, Oracle)
	□ Collaborative applications/groupware (e.g., Slack, MS Teams, Zoom, Webex)
	□ Virtual boardroom (e.g., Sherpany, Dilligent Boards, iDeals, BoardEffect)
	□ Cloud storage/computing (e.g., AWS, MS Azure, IBM Cloud, Google Cloud)
	□ Networking and control of machines and plants via the Internet (cyber-physical systems)
	□ Internet of Things (e.g., IoT-sensors, RFID chips, e-grains, NFC)
	□ Robotics, automated transport or production systems (e.g., industrial, service or mobile robots, drones)
	□ Additive manufacturing processes (e.g., 3D printing)
	□ Virtual/augmented reality (e.g., MS HoloLens)
	□ Blockchain (distributed ledger technology)
Does your company apply regular evaluations of your employees' performances carried out by one or more supervisors at least once a year?	
Management staff	□ Yes, where the share amounts to __ %<br>□ No
Employees without management responsibilities	□ Yes, where the share amounts to __ %<br>□ No

Continued on the next page...

...Table A12 continued

Survey question	Response options
For whom do you apply performance targets?	
Management staff	☐ Yes, where the share amounts to __ %<br>☐ No
Employees without management responsibilities	☐ Yes, where the share amounts to __ %<br>☐ No
What percentage of employees usually receive performance pay in your company? Please distinguish between employees at different hierarchical levels.	
Top management	☐ Yes, where the share amounts to __ %<br>☐ No
Low and middle management	☐ Yes, where the share amounts to __ %<br>☐ No
Non-managerial employees	☐ Yes, where the share amounts to __ %<br>☐ No

References

Acemoglu, D., Aghion, P., Lelarge, C., Van Reenen, J., and Zilibotti, F. (2007). Technology, Information, and the Decentralization of the Firm. *The Quarterly Journal of Economics*, 122(4):1759–1799.

Acemoglu, D., Autor, D., Dorn, D., Hanson, G. H., and Price, B. (2016). Import Competition and the Great US Employment Sag of the 2000s. *Journal of Labor Economics*, 34(S1):141–198.

Acemoglu, D. and Restrepo, P. (2018). The Race between Man and Machine: Implications of Technology for Growth, Factor Shares, and Employment. *The American Economic Review*, 108(6):1488–1542.

Acemoglu, D. and Restrepo, P. (2020). Robots and Jobs: Evidence from US Labor Markets. *Journal of Political Economy*, 128(6):2188–2244.

Adepoju, O. (2022). 3D Printing/Additive Manufacturing. In Adepoju, O., Aigbavboa, C., Nwulu, N., and Onyia, M., editors, *Re-Skilling Human Resources for Construction 4.0: Implications for Industry, Academia and Government*, pages 117–139. Springer International Publishing, Cham.

Alade, S. M. (2023). Design and Implementation of a Web-based Document Management System. *International Journal of Information Technology and Computer Science*, 15(2):35–53.

Aral, S., Brynjolfsson, E., and Wu, D. J. (2006). Which Came First, IT or Productivity? Virtuous Cycle of Investment and Use in Enterprise Systems. In *Proc. 27th Annual Internat. Conf. Inform. Systems*, pages 1819–1839, Milwaukee.

Aral, S., Brynjolfsson, E., and Wu, L. (2012). Three-Way Complementarities: Performance Pay, Human Resource Analytics, and Information Technology. *Management Science*, 58(5):913–931.

Arena, F., Collotta, M., Pau, G., and Termine, F. (2022). An Overview of Augmented Reality. *Computers*, 11(2):28.

Arntz, M., Gregory, T., and Zierahn, U. (2016). The Risk of Automation for Jobs in OECD Countries: A Comparative Analysis. Technical Report 189, OECD Publishing, Paris.

Autor, D. and Salomons, A. (2018). Is Automation Labor Share-Displacing? Productivity Growth, Employment, and the Labor Share. *Brookings Papers on Economic Activity*, 2018(1):1–63.

Autor, D. H. (2015). Why Are There Still So Many Jobs? The History and Future of Workplace Automation. *Journal of Economic Perspectives*, 29(3):3–30.

Autor, D. H., Katz, L. F., and Kearney, M. S. (2006). The Polarization of the U.S. Labor Market. *The American Economic Review*, 96(2):189–194.

Bayo-Moriones, A., Erro-Garcés, A., and Lera-López, F. (2022). Computer Use and Pay for Performance. *Human Resource Management Journal*, 32(2):341–363.

Beckmann, M. and Gerten, E. (2018). Die Entwicklung der Arbeit in Zeiten der Digitalisierung. In *Schulthess Manager Handbuch 2018/2019*, pages 209–217. Schulthess Verlag, Zürich.

Brau, R. I., Sanders, N. R., Aloysius, J., and Williams, D. (2023). Utilizing People, Analytics, and AI for Decision Making in the Digitalized Retail Supply Chain. *Journal of Business Logistics*, 45:e12355.

Brynjolfsson, E. and Mitchell, T. (2017). What Can Machine Learning Do? Workforce Implications. *Science*, 358(6370):1530–1534.

Calvino, F., Criscuolo, C., Marcolin, L., and Squicciarini, M. (2018). A Taxonomy of Digital Intensive Sectors. OECD Science, Technology and Industry Working Papers 2018/14, OECD.

Casino, F., Dasaklis, T. K., and Patsakis, C. (2019). A Systematic Literature Review of Blockchain-Based Applications: Current Status, Classification and Open Issues. *Telematics and Informatics*, 36:55–81.

Cho, W., Choi, S., and Choi, H. (2023). Human Resources Analytics for Public Personnel Management: Concepts, Cases, and Caveats. *Administrative Sciences*, 13(2):41.

Collazos, C. A., Gutiérrez, F. L., Gallardo, J., Ortega, M., Fardoun, H. M., and Molina, A. I. (2019). Descriptive Theory of Awareness for Groupware Development. *Journal of Ambient Intelligence and Humanized Computing*, 10(12):4789–4818.

Dixon, J., Hong, B., and Wu, L. (2021). The Robot Revolution: Managerial and Employment Consequences for Firms. *Management Science*, 67(9):5586–5605.

Felice, G., Lamperti, F., and Piscitello, L. (2022). The Employment Implications of Additive Manufacturing. *Industry and Innovation*, 29(3):333–366.

Ferreira, P., Meirinhos, V., Rodrigues, A. C., and Marques, A. (2021). Virtual and Augmented Reality in Human Resource Management and Development: A Systematic Literature Review. *IBIMA Business Review*, pages 1–18.

Frank, R., Schumacher, G., and Tamm, A. (2023). Cloud Transformation – How the Public Cloud is Changing Businesses. In Frank, R., Schumacher, G., and Tamm, A., editors, *Cloud Transformation: The Public Cloud Is Changing Businesses*, pages 247–263. Springer Fachmedien, Wiesbaden.

Funk, M. J., Westreich, D., Wiesen, C., Stürmer, T., Brookhart, M. A., and Davidian, M. (2011). Doubly Robust Estimation of Causal Effects. *American Journal of Epidemiology*, 173(7):761–767.

Gaur, B., Shukla, V. K., and Verma, A. (2019). Strengthening People Analytics through Wearable IOT Device for Real-Time Data Collection. In *International Conference on Automation, Computational and Technology Management (ICACTM)*, pages 555–560, London.

Gelbard, R., Ramon-Gonen, R., Carmeli, A., Bittmann, R. M., and Talyansky, R. (2018). Sentiment Analysis in Organizational Work: Towards an Ontology of People Analytics. *Expert Systems*, 35(5):e12289.

Giermindl, L. M., Strich, F., Christ, O., Leicht-Deobald, U., and Redzepi, A. (2022). The Dark Sides of People Analytics: Reviewing the Perils for Organisations and Employees. *European Journal of Information Systems*, 31(3):410–435.

Hannola, L., Richter, A., Richter, S., and Stocker, A. (2018). Empowering Production Workers With Digitally Facilitated Knowledge Processes – A Conceptual Framework. *International Journal of Production Research*, 56(14):4729–4743.

Helper, S. and Henderson, R. (2014). Management Practices, Relational Contracts, and the Decline of General Motors. *Journal of Economic Perspectives*, 28(1):49–72.

Hitt, L. M., Wu, D., and Zhou, X. (2002). Investment in Enterprise Resource Planning: Business Impact and Productivity Measures. *Journal of Management Information Systems*, 19(1):71–98.

Katuse, P. and Gaur, D. (2024). Role of Talent Management in People Analytics and Industry 4.0. In Kumar Shukla, V., Kulkarni, P., Gaur, D., Pradeep, N., Lacap, J. P. G., and Omrane, A., editors, *Industry 4.0 and People Analytics*, pages 311–322. Apple Academic Press, New York, 1 edition.

Koriat, N. and Gelbard, R. (2019). Knowledge Sharing Analytics: The Case of IT Workers. *Journal of Computer Information Systems*, 59(4):308–318.

Lee, J., Lapira, E., Bagheri, B., and Kao, H. (2013). Recent Advances and Trends in Predictive Manufacturing Systems in Big Data Environment. *Manufacturing Letters*, 1(1):38–41.

Manski, S. (2017). Building the Blockchain World: Technological Commonwealth or Just More of the Same? *Strategic Change*, 26(5):511–522.

Nyman, S., Bødker, M., and Blegind Jensen, T. (2024). Reforming work patterns or negotiating workloads? Exploring alternative pathways for digital productivity assistants through a problematization lens. *Journal of Information Technology*, 39(3):503–520.

Pistorius, J. (2020). *Industrie 4.0 – Schlüsseltechnologien für die Produktion: Grundlagen • Potenziale • Anwendungen*. Springer Vieweg, Berlin/Heidelberg, 1 edition.

Polzer, J. T. (2022). The Rise of People Analytics and the Future of Organizational Research. *Research in Organizational Behavior*, 42:100181.

SapienceAnalytics (2021). The Impact of Valuing Employee Effort [White Paper]. https://sapienceanalytics.com/wp-content/uploads/2022/01/WP-The-Impact-of-Valuing-Employee-Effort-1.pdf.

Solon, G., Haider, S. J., and Wooldridge, J. M. (2015). What Are We Weighting For? *Journal of Human Resources*, 50(2):301–316.

Tambe, P., Cappelli, P., and Yakubovich, V. (2019). Artificial Intelligence in Human Resources Management: Challenges and a Path Forward. *California Management Review*, 61(4):15–42.

Waschull, S., Bokhorst, J. A. C., Molleman, E., and Wortmann, J. C. (2020). Work Design in Future Industrial Production: Transforming Towards Cyber-Physical Systems. *Computers & Industrial Engineering*, 139:105679.

Windelband, L. (2014). Zukunft der Facharbeit im Zeitalter ,,Industrie 4.0“. *Journal of Technical Education*, 2(2):138–160.
